# Understanding Intimate Partner Violence Through Police Crime Data: Descriptive and Temporal Insights

**DOI:** 10.3390/bs16010048

**Published:** 2025-12-25

**Authors:** Charmayne Mary Lee Hughes

**Affiliations:** Age-Appropriate Human-Machine Systems, Institute of Psychology and Ergonomics, Technische Universität Berlin, Fasanenstraße 1, 10623 Berlin, Germany; hughes@tu-berlin.de; Tel.: +49-30-31-41900-2002

**Keywords:** intimate partner violence, crime reporting, police data, violence, domestic violence

## Abstract

Police crime reports are a critical but often underutilized source of information for understanding intimate partner violence (IPV). They provide systematic, population-level data on when, where, and how IPV incidents occur, complementing surveys and clinical studies. This study provides a descriptive analysis of IPV crime reports in Los Angeles (January 2020–December 2023) and models temporal trends using Seasonal Autoregressive Integrated Moving Average (SARIMA). Results showed that a total of 74,776 IPV-related incidents were reported to the LAPD in the four-year period, averaging 51.22 incidents per day (SD = 10.84). Most incidents occurred in residential settings (71.9%), followed by public spaces (18.2%) and transportation settings (6.5%). Females accounted for the majority of incidents (77.35%) compared to males (22.65%), and Physical IPV was the most frequently reported subtype (77.0%). Of these Physical IPV reports, most incidents did not involve a weapon (83.82%), while the use of firearms, bladed weapons, blunt objects, and improvised implements was relatively uncommon. Temporal modeling using SARIMA indicated that month-to-month variation was dominated by stable seasonal and autoregressive dynamics, with no evidence of a distinct pandemic-specific shift in call volume. By integrating descriptive and temporal analyses, the study offers actionable insights for public health, law enforcement, and community organizations working to prevent and respond to IPV.

## 1. Introduction

Intimate partner violence (IPV) is a pervasive and complex public health problem, affecting individuals across all ages, genders, and socioeconomic groups (Breiding et al., 2015; Gracia-Moreno et al., 2005; Xiong et al., 2025). Rather than occurring as isolated incidents, IPV often unfolds in escalating patterns that can persist over time, leading to profound and long-lasting consequences for survivors’ health, well-being, and social functioning (Gracia-Moreno et al., 2005; Xiong et al., 2025). The impacts extend beyond immediate physical harm, contributing to chronic medical conditions, heightened risk of mental health disorders (J. C. Campbell, 2002), and disruptions in social and familial relationships. Moreover, IPV generates intergenerational effects, influencing not only survivors but also their families and broader communities (Ehrensaft et al., 2017).

Much of the scientific evidence on IPV is derived from community-based surveys (Breiding et al., 2015), healthcare or clinical settings (Loder & Momper, 2020; Jónasdóttir et al., 2022; Mariscal et al., 2020), and specialized facilities such as shelters, correctional institutions, or social service agencies (Kennedy & Mennicke, 2018; L. B. Klein et al., 2021). While these sources provide valuable insights into prevalence, risk factors, and outcomes, they represent only a portion of IPV experiences. As with most research on IPV, findings are influenced by underreporting (Bergenfeld et al., 2025), often due to recall bias in surveys or survivors’ reluctance to disclose experiences with service providers (Lohr, 2019). Consequently, important aspects of the full scope, temporal dynamics, and contextual factors of reported IPV remain insufficiently understood.

Police crime reports are a valuable but often underutilized source of information on reported IPV incidents (Henning et al., 2021; A. R. Klein, 2009). Unlike surveys or clinical data, these records provide systematic, population-level information on when and where incidents occur, who is involved, and the surrounding context. Depending on the police department, records may include calls-for-service documenting requests for assistance; crime or incident reports detailing investigated offenses; and arrest records capturing apprehensions and charges. Supplemental sources, such as citations, field interviews, and evidence logs, may provide additional context. It is important to note, however, that police data capture only reported incidents and cannot overcome the general under-reporting of IPV. Many cases never come to the attention of authorities due to reluctance to disclose, social stigma, or fear of retaliation. Therefore, while police records are useful for analyzing temporal and spatial patterns of reported IPV, they do not provide a complete picture of the true incidence in the population.

Despite these limitations in capturing the full scope of IPV, police-reported data have been widely used to study a range of other crimes (Dau et al., 2023; Hu et al., 2018), including assaults, property offenses, and gang-related activity, offering insights into temporal and spatial patterns, survivor and offender characteristics, and risk factors. For example, Kaplan et al. (2023) analyzed rape reports and clearance data from multiple California law enforcement agencies (2013–2016) and found that higher female officer representation was linked to increased rape reporting but lower clearance rates, suggesting that departmental composition can shape patterns of crime reporting and investigation. This example demonstrates how police records can reveal reporting trends and inform targeted interventions. Extending similar approaches to IPV could deepen understanding and complement survey and clinical evidence.

Although reporting to law enforcement represents just one avenue for help-seeking, police reports remain critical for understanding patterns of IPV reporting within a community. Examining police-reported incidents over time can reveal temporal and seasonal patterns (Miller et al., 2022, 2024; Sanz-Barbero et al., 2018), which are essential for supporting evidence-based policy and evaluating interventions. For this study, data were obtained from the Los Angeles Open Data Portal (Los Angeles City, 2025), which provides publicly accessible, anonymized crime and incident reports. Open data initiatives like this promote transparency, accountability, and public engagement, enabling researchers, policymakers, and community organizations to monitor trends, identify high-risk areas, and inform prevention and support strategies.

To capitalize on the insights offered by police-reported IPV data, this study aims to characterize reported IPV incidents in Los Angeles from January 2020 to December 2023. It examines survivor demographic factors (biological sex and age), incident location, IPV subtype, and, for Physical IPV incidents, the type of weapon used. Temporal trends in reported IPV are examined descriptively by year, month, and day of the week, and further explored using Seasonal Autoregressive Integrated Moving Average (SARIMA) time-series modeling to account for seasonality and temporal dependence. By achieving these aims, the study provides information to inform public health practice, law enforcement, and community-based support, while acknowledging that the findings reflect patterns in reported incidents, not true underlying IPV prevalence.

## 2. Methods

### 2.1. LAPD Structure and Crime Reporting

The Los Angeles Police Department (LAPD) is the third-largest municipal police force in the United States (Los Angeles Police Department, 2024), employing approximately 12,000 personnel, including roughly 9992 sworn and municipal officers and 3042 civilian personnel (Los Angeles Police Department, 2020). The department serves a diverse population of approximately four million persons (Los Angeles City Planning, 2020) across a 503-square-mile jurisdiction, encompassing a wide range of geographical terrain, from densely populated urban centers and sprawling residential neighborhoods to hills, canyons, and industrial zones.

Reported crimes to the LAPD typically originate from calls-for-service made by the public. While multiple calls may relate to the same event, only one verified incident is recorded in the reported crime data, reducing the noise inherent in raw calls-for-service (Klinger & Bridges, 1997). In short, calls-for-service reflect public requests for police assistance and indicate broader patterns of police demand, fear of crime, and potential harm, whereas reported crimes represent verified incidents retained in the official records (Kahmann et al., 2022). Verified incidents are first handled by uniformed patrol officers in one of the LAPD’s 21 divisions, who prepare the initial report based on statements from survivors and witnesses, including suspect descriptions. The case is then assigned to a detective in the relevant division (e.g., Vice, Robbery–Homicide, Narcotics), who conducts follow-up investigations, pursues leads, and interviews survivors, witnesses, and suspects as appropriate.

### 2.2. Data Source

Criminal incident-level data on LAPD IPV-related reports were obtained from the publicly available LA Open Data portal (Los Angeles City, 2025). The dataset includes detailed information on crime type, date and time of occurrence, geographic coordinates, reporting district, and whether incidents occurred within one of the 21 Geographic Areas (Patrol Divisions) of Los Angeles. Additional variables include survivor characteristics (biological sex, age, and descent^1^), incident location, type of weapon used, and the status of each incident.

### 2.3. Case Definition

For this study, data were restricted to reported crimes involving adults (defined as individuals aged 18 years and older) with a known biological sex (i.e., male, female) between 1 January 2020, and 31 December 2023^2^.

IPV cases were identified using Modus Operandi (MO) and Crime Type codes. Incidents were classified as IPV if the MO code was 2000 (Domestic Violence); the Crime Type code was 236 (Spousal/Cohabitant Abuse–Felony) or 626 (Spousal/Cohabitant Abuse–Misdemeanor); or the MO code indicated the perpetrator was an intimate partner (1813: current/former spouse, 1814: current/former boyfriend/girlfriend, 1819: common-law spouse, 1821: spouse, 1823: brief encounter/date) and the MO or Crime Type code corresponded to an IPV-related offense (e.g., 0416: hit with weapon; 0417: kicked; 0421: threaten to kill; 0430: individual shot; 763: stalking; 815: sexual penetration with a foreign object; 810: unlawful sex; 230: assault with a deadly weapon/aggravated assault; 624: battery/simple assault; 930: criminal threats without weapon displayed).

Several categories of incidents were subsequently removed to ensure the analytic sample included only IPV cases. Criminal homicide was excluded because it represents a qualitatively distinct outcome with different legal, clinical, and epidemiological implications. Child-directed offenses (e.g., physical child abuse, lewd acts with a child) were excluded because the survivor was not an intimate partner. Categories of offenses unrelated to IPV dynamics (e.g., pickpocketing, traffic violations, or replica firearm sales and manufacture, human trafficking) were also excluded, as they do not reflect violence or abuse within an intimate relationship.

To enhance reliability, all IPV cases identified through these codes were independently reviewed by a second coder with expertise in IPV research. Discrepancies between coders were resolved through consensus, ensuring consistent classification and reducing the risk of misclassification. While this dual-coding approach strengthens confidence in the validity of case definitions, it is important to note that administrative police data inherently underreport IPV and may omit forms such as psychological, economic, or coercive abuse.

### 2.4. Incident Classification and Characteristics

Survivor age was recorded in years and, for this study, categorized into ten discrete age bands: 18–24, 25–29, 30–34, 35–39, 40–44, 45–49, 50–54, 55–59, 60–64, and 65 years or older. Incident locations were categorized according to the premise where the event occurred, grouped into nine categories: Residential, Transportation, Retail, Entertainment, Education, Health & Care, Religious, Institutional/Public Services, and Other. Residential included private living spaces (homes, apartments, transitional housing). Transportation covered public and private transit, streets, freeways, and parking lots. Retail encompassed markets, department stores, and other commercial establishments. Entertainment included movie theatres, nightclubs, and sports venues. Education comprised schools, colleges, universities, and vocational/trade schools. Health & Care included hospitals, medical offices, nursing homes, and fitness facilities. Religious encompassed churches, synagogues, mosques, and other places of worship. Institutional/Public Services included government buildings, police facilities, post offices, and libraries. Other included unknown or unspecified locations.

After exclusions, IPV incidents were categorized into five subtypes: the four traditionally recognized forms outlined by the CDC (physical, sexual, psychological, and stalking; Breiding et al., 2015) and economic IPV to capture financial and material abuse (Postmus et al., 2020). Physical IPV (Esquivel-Santoveña & Dixon, 2012) included the use or threat of force intended to cause bodily harm, such as assault, battery, or brandishing weapons. Sexual IPV (Barker et al., 2019) encompassed unwanted sexual contact or coercion, including rape, attempted rape, sexual penetration with a foreign object, sodomy, oral copulation, and pandering. Psychological IPV (Carney & Barner, 2012) involved behaviors intended to cause emotional distress, fear, or loss of autonomy, such as threats, harassment, intimidation, false imprisonment, violations of restraining orders, cruelty to animals, arson, and other forms of coercive control. Stalking (Logan & Walker, 2009) was defined as repeated or unwanted surveillance or harassment, including prowling, trespassing, and peeping. Economic IPV reflected financial exploitation, resource deprivation, and material control (Meyer et al., 2024; Postmus et al., 2020) and was operationalized in this dataset as robbery, burglary, theft, fraud, and extortion.

Weapons used in the commission of Physical IPV included no weapon, firearms, blunt objects, bladed weapons, chemical agents, miscellaneous or improvised weapons, and other weapons. Incidents involving only bodily force (hands, fists, feet, or other parts of the body) were coded as no weapon. Firearms encompassed manufactured weapons designed to discharge bullets (e.g., handguns, rifles, pistols, and shotguns). Blunt objects (e.g., hammers, clubs, and pipes) were used to strike survivors. Bladed weapons (e.g., knives, swords, daggers, and machetes) were designed for cutting or stabbing. Chemical agents (e.g., pepper spray or caustic substances) were used to harm or incapacitate survivors. Miscellaneous or improvised weapons referred to everyday items not originally designed to injure but weaponized in IPV contexts, including bottles, screwdrivers, rocks, ropes, and vehicles. Other weapons referred to items outside of these categories, such as explosive devices.

### 2.5. Data Analysis

#### 2.5.1. Case Report Descriptives

Descriptive statistics were calculated to summarize the distribution of IPV cases by survivor age and biological sex, incident location, IPV subtype, and weapon type for Physical IPV cases. Chi-square goodness-of-fit tests were used to assess whether observed distributions differed from uniform expectations. Temporal trends in IPV reporting were examined by year, month, and day of the week. Differences in daily case counts across these categories were initially tested using Kruskal–Wallis nonparametric tests, appropriate for skewed count data that do not meet normality assumptions, though these tests do not account for temporal autocorrelation. Where overall tests were significant, pairwise comparisons were conducted using Dunn’s test with Bonferroni correction. *p* values of less than 0.05 were considered to be statistically significant. These exploratory analyses provided initial insights into seasonal and weekly variation, which were subsequently modeled using SARIMA to account for temporal dependence and seasonality.

#### 2.5.2. Time Series Analysis

Time series analysis began by examining the stationarity of the monthly IPV case counts using the Augmented Dickey–Fuller (ADF; Dickey & Fuller, 1979) and Kwiatkowski-Phillips-Schmidt-Shin (KPSS; Kwiatkowski et al., 1992) tests. A *p*-value less than 0.05 from the ADF test indicates rejection of the null hypothesis of non-stationarity, providing evidence that the series is stationary. For the KPSS test, a *p*-value greater than 0.05 indicates failure to reject the null hypothesis of stationarity. Autocorrelation and partial autocorrelation functions were then inspected to identify potential temporal dependencies (Box et al., 2015). Variance decomposition was performed to quantify the contributions of residual, trend, and seasonal components to the overall variance in monthly case counts.

For modeling, a SARIMA framework was applied (Box et al., 2015). SARIMA extends the traditional Autoregressive Integrated Moving Average model (ARIMA; Box & Jenkins, 1976) by incorporating repeating seasonal patterns, such as monthly or yearly cycles. Initial differencing terms (*d* and *D*) were estimated using an automated SARIMA procedure (auto_arima). Candidate ranges for non-seasonal (*p*, *q*) and seasonal (*P*, *Q*) parameters were defined based on auto_arima estimates to guide a combinatorial grid search, assuming monthly seasonality (*m* = 12). The best-fitting model was selected based on the lowest Akaike Information Criterion (AIC; Akaike, 1974), balancing model fit and complexity.

Residuals from the final model were evaluated using autocorrelation function (ACF) and partial autocorrelation function (PACF) plots. Model adequacy was further assessed using the Ljung–Box test for residual autocorrelation (Ljung & Box, 1978), the Jarque–Bera test for normality of residuals (Jarque & Bera, 1987), and a Goldfeld–Quandt-style test for heteroskedasticity (Goldfeld & Quandt, 1965) in order to evaluate variance stability. Forecasts for the subsequent 12 months were generated, with confidence intervals reflecting model uncertainty and assuming normally distributed residuals.

## 3. Results

### 3.1. Case Report Descriptives

A total of 74,776 IPV-related incidents reported to the LAPD were documented during the study period, averaging 51.22 per day (SD = 10.84). Descriptive statistics are presented in Table 1. Females accounted for the majority of incidents compared to males (77.35% vs. 22.65%), χ^2^(1, *N* = 74,776) = 22,370.9, *p* < 0.001. Significant differences were observed across age groups, χ^2^(9, *N* = 74, 776) = 33,110, *p* < 0.001, with the largest proportions observed in the 18–24 (17.10%), 25–29 (19.49%), and 30–34 (18.68%) year groups, and the smallest proportions in the 60–64 (1.96%) and 65+ (1.90%) year groups.

IPV reports differed significantly by location, χ^2^(10, *N* = 74,789) = 381,910.6, *p* < 0.001. Most incidents occurred in residential settings (71.9%), followed by public spaces (18.2%) and transportation settings (6.5%). Smaller proportions were reported in business locations, retail, institutional/public services, health and care facilities, recreation/entertainment venues, educational institutions, and religious premises.

Physical IPV was the most frequently reported subtype (77.0%), followed by Psychological IPV (15.84%), Economic IPV (4.76%), Sexual IPV (1.79%), and stalking (0.59%), χ^2^(4, *N* = 74,789) = 157,345.2, *p* < 0.001. Among reports of Physical IPV, most incidents did not involve a weapon (83.82%). The use of firearms (2.25%), bladed weapons (3.98%), blunt objects (1.87%), and improvised implements (7.09%) were comparatively rare, χ^2^(6, *N* = 57,493) = 228,302.8, *p* < 0.001.

### 3.2. Temporal Trends

To establish baseline patterns, IPV reports were first summarized by year, month, and day of week. Yearly averages showed a relatively stable volume of daily reports, with means ranging from 49.41 (SD = 10.42) in 2023 to 52.28 (SD = 11.55) in 2022. A Kruskal–Wallis test indicated significant differences across years, *H* = 13.858, *p* = 0.0031. Post hoc comparisons revealed that 2023 had significantly fewer daily reports than 2021 and 2022 (both *p*’s < 0.01), whereas 2020 did not differ significantly from 2021 or 2022 (*p* > 1).

Monthly summaries showed significant seasonal variation (Kruskal–Wallis H = 95.097, *p* < 0.001). Reports peaked in July (M = 1710.25, SD = 105.94) and August (M = 1721.50, SD = 66.66), and were lowest in February (M = 1399.25, SD = 109.80). Post hoc comparisons indicated that July and August were significantly higher than the first four months and last two months of the year (all *p*’s < 0.05), while February and December were significantly lower than the fifth through eight calendar months (all *p*’s < 0.05).

Daily IPV reports varied significantly across weekdays (*H* = 392.83, *p* < 0.001). Post hoc comparisons showed that Saturday (M = 56.66, SD = 9.78) and Sunday (M = 62.80, SD = 10.59) had significantly higher counts than weekdays (M = 46.01–50.26, all *p*’s < 0.001). Friday (M = 50.26, SD = 9.05) and Monday (M = 49.69, SD = 9.78) did not differ from each other but were lower than weekend days, while mid-week days, Tuesday (M = 46.21, SD = 8.50), Wednesday (M = 46.01, SD = 7.50), and Thursday (M = 46.83, SD = 9.05), had the lowest counts and did not differ significantly from one another (all *p*’s > 1.0). Overall, these results indicate a consistent pattern of increased IPV reporting on weekends and lower reporting during mid-week.

### 3.3. Time-Series Analysis

Descriptive analyses revealed meaningful fluctuations in IPV-related police reports across years, months, and weekdays, with peaks on weekends and during summer months, motivating the use of SARIMA models to capture underlying seasonal patterns and temporal dependencies. Stationarity analysis of the monthly IPV reports yielded an ADF statistic of −1.091 (*p* = 0.719; critical values: 1% = −3.610, 5% = −2.939, 10% = −2.608), indicating non-stationarity according to this test. In contrast, the KPSS statistic was 0.071 (*p* = 0.10; critical values: 10% = 0.347, 5% = 0.463, 2.5% = 0.574, 1% = 0.739), suggesting that the series can be considered stationary. The conflicting results point to the presence of mild trends or low-level non-stationarity, highlighting the importance of applying appropriate differencing and seasonal adjustments in subsequent modeling to ensure valid forecasts and accurate parameter estimation.

**Figure 1:**
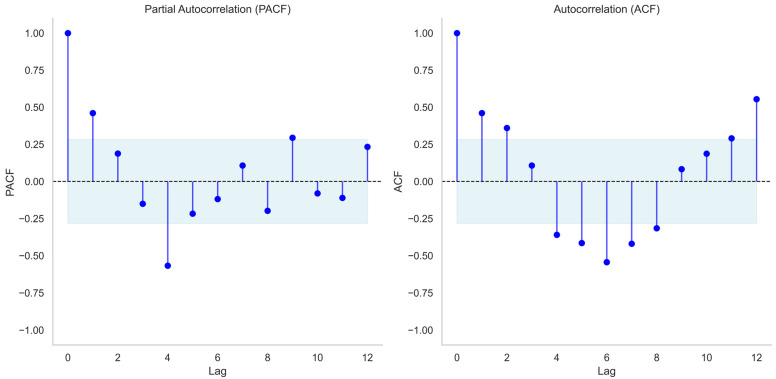
Autocorrelation function (ACF) and partial autocorrelation function (PACF) of monthly IPV reports up to 12 lags. Dashed lines indicate significance bounds at 95%.

Variance decomposition of the monthly IPV reports using Seasonal-Trend decomposition using LOESS (STL) indicated that the seasonal component contributed the largest share of the total variance, followed by the trend and residual components. Specifically, the seasonal component accounted for 81.48% of the total variance, the trend for 10.55%, and the residual for 6.82%, highlighting that seasonality is the dominant driver of variation in the observed series. Autocorrelation and partial autocorrelation analyses further illustrated temporal dependencies in the data (Figure 1). The ACF showed moderate positive correlations at lag 1 (0.461) and lag 2 (0.360), followed by negative correlations at intermediate lags (e.g., –0.360 at lag 4 and –0.544 at lag 6) and a strong resurgence at lag 12 (0.555), consistent with annual seasonality. The PACF indicated a sharp negative correlation at lag 4 (–0.568), with smaller fluctuations at other lags. These patterns suggest both short-term autocorrelation and seasonal effects, supporting the use of autoregressive and seasonal components in subsequent time series modeling.

The optimal seasonal ARIMA model identified for the series was ARIMA(1,0,2)(1,0,0)_[12]_ with an intercept, fitted to 48 monthly observations from January 2020 to December 2023. Parameter estimates indicated a significant intercept (818.95, *p* = 0.001) and first-order autoregressive term (AR1 = −0.949, *p* < 0.001), and a seasonal AR term at lag 12 (AR.S12 = 0.727, *p* < 0.001). The second moving average term (MA2 = 0.369) was positive but not statistically significant (*p* = 0.157). The residual variance (σ^2^) was estimated at 5752.38. The information criteria for this model were AIC = 572.43 and BIC = 583.65.

Residual diagnostics suggested that the model adequately captured the main trend and seasonal fluctuations of the series. The Ljung–Box test (Q1 = 0.57, *p* = 0.45) indicated no significant short-term autocorrelation, suggesting that residuals behave like white noise. The Jarque–Bera test (JB = 3.41, *p* = 0.18) suggested approximate normality of residuals, and the heteroskedasticity test (H = 1.27, *p* = 0.64) confirmed stable variance over time.

**Figure 2:**
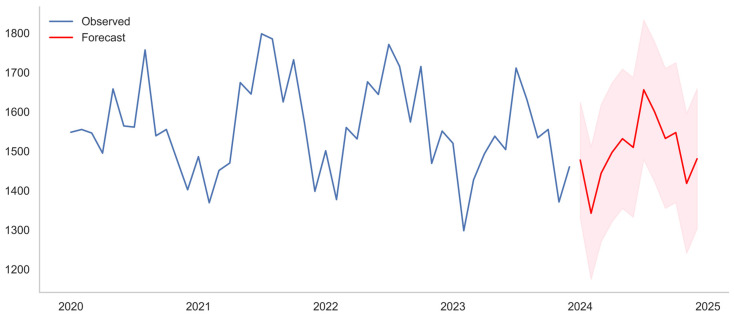
Monthly IPV reports to the Los Angeles Police Department. Blue lines represent observed data from January 2020 to December 2023. The red line shows ARIMA(1,0,2)(1,0,0)_[12]_ model forecasts for January–December 2024, with the pink shaded area indicating the 95% confidence intervals.

The fitted model was then applied to generate a 12-month forecast (1 January 2024 through 31 December 2024), with 95% confidence intervals quantifying prediction uncertainty. The projections indicate that monthly IPV reports are expected to remain roughly stable, with some months slightly lower than previous years, while retaining the seasonal fluctuations evident in the observed data (Figure 2). Forecasted values suggest moderate month-to-month variability, with anticipated peaks aligning with seasonal highs documented in prior years.

## 4. Discussion

This study analyzed IPV-related crimes reported to the LAPD from January 2020 to December 2023, corroborating previously established IPV characteristics while revealing novel insights into this pervasive form of violence. The following main findings were observed. First, young adults and women accounted for the majority of survivors, and most incidents occurred in residential locations. Second, most reports involved physical IPV, with the majority of cases not involving a weapon. Third, police-reported incidents peaked during summer and on weekends, consistently across years. The discussion focuses primarily on these main findings.

Concurrent with prior literature across multiple settings (A. M. Campbell et al., 2020; Mariscal et al., 2020), police-reported IPV was most prevalent among younger adults, particularly those in their 20s and early 30s, while older adults were less frequently represented. Women accounted for the majority of police-reported IPV cases (77.4%), with men representing 22.7% of cases. Although men represented a smaller proportion of cases, it is important to consider the unique dynamics affecting male survivors. Research consistently shows that men are generally less likely to seek help for IPV (cf. Machado et al., 2024), whether from informal sources such as friends and family or from formal sources such as support services or law enforcement. When male survivors do contact the police, they often face additional barriers, including ridicule, skepticism, indifference, or even being falsely accused of perpetration or arrested (Dim & Lysova, 2021; Lysova, 2025), which can exacerbate trauma and discourage future reporting. The observed percentage of male survivors in Los Angeles may reflect not only greater willingness among men to seek assistance but also cases initiated by third parties (e.g., neighbors, relatives, or bystanders). This highlights the importance of considering both reporting behaviors and the broader social and institutional context when interpreting police-reported IPV data.

Most police-reported IPV incidents in this study occurred in residential settings (71.9%), underscoring the home as the main site of harm. Smaller proportions occurred in public spaces (18.2%), transportation settings (6.5%), and other non-residential venues. These findings align with earlier work showing that IPV most often takes place in private dwellings, typically the survivor’s home (Greenfeld, 1998; Dobash & Dobash, 1984). The proportion of public space incidents (18.2%) is comparable to prior estimates, with Greenfeld (1998) reporting 15% and Dobash and Dobash (1984) finding 13% of self-reported events and 19% of official police records. Rennison and Welchans (2000) further noted the heightened risks of public spaces, where 8% of female survivors and 11% of male survivors were killed. Overall, these results reinforce the centrality of residential environments in police-reported IPV while underscoring the persistent dangers of public settings.

The study also found that the majority of police-reported IPV involved physical violence, a result consistent with prior research (Bonomi et al., 2006; Voce & Boxall, 2018). Physical IPV frequently produces visible, tangible harm (e.g., bruises, cuts, or property damage) that can be readily documented and often meets statutory definitions of assault. In contrast, Psychological IPV, while often the most common form of IPV (Breiding et al., 2015), is underrepresented in police-reported data. This underreporting likely reflects multiple factors: survivors may not perceive non-physical abuse as criminal, find it difficult to document or prove, or anticipate limited intervention by authorities. Consequently, police involvement for Psychological IPV generally occurs only when the abuse reaches a level of severity at which the survivor perceives law enforcement as the only available option to escape further harm (Duterte et al., 2008).

Within these reported incidents, the majority did not involve weapons, with firearms, bladed weapons, blunt objects, and improvised implements used relatively rarely. These findings align with prior research indicating that IPV is most often enacted through direct bodily force (i.e., via the hands, fists, and feet), while other weapons represent only a minority of reported cases (Addington & Perumean-Chaney, 2014; A. M. Campbell et al., 2020; Joshi & Sorenson, 2010; Truman & Morgan, 2014). For example, National survey data indicate that roughly one in five non-lethal IPV incidents involves a weapon, most commonly firearms or knives, though a wide variety of other objects (from household items to sports equipment) have also been used (Truman & Morgan, 2014).

Non-physical forms of IPV, including Psychological and Sexual IPV, as well as Economic IPV, are frequently underreported, reflecting social normalization, stigma, and the difficulty of substantiating harm. Stalking was also rarely reported, likely due to its covert, prolonged nature (Spitzberg & Cupach, 2007), police classification practices (Brady & Nobles, 2017), and the legal requirement to prove beyond a reasonable doubt that the defendant willfully and maliciously followed or harassed the person and made a credible threat intended to place the person in reasonable fear for their safety (Brady & Reyns, 2020). Collectively, these findings suggest that police-reported data disproportionately capture acute, visible harm, while more subtle or chronic forms of IPV remain underrepresented.

The seasonal dynamics of police-reported IPV between 2020 and 2023 reveal a consistent cyclical pattern, with peaks in the summer months (particularly July and August), and troughs in February. Beginning in March 2020, Los Angeles implemented multiple phases of COVID-19 restrictions, including stay-at-home orders, curfews, business and school closures, and limits on social gatherings, which altered daily life and could have influenced household stress, interpersonal dynamics, and reporting behavior. Time-series analysis indicates that month-to-month variation was dominated by stable seasonal and autoregressive dynamics, with no evidence of a distinct pandemic-specific shift in case volume. This suggests that the temporal rhythms driving higher police-reported IPV in warmer months remained a powerful and stable influence, even amid the broader context of pandemic-related disruptions (Leslie & Wilson, 2020; Miller et al., 2022, 2024).

These findings are largely consistent with broader criminological evidence demonstrating that IPV generally peaks in summer months (A. M. Campbell et al., 2020; Gracia et al., 2025; Michael & Zumpe, 1986) and during periods of extreme heat, such as heat waves (Allen et al., 2021; Sanz-Barbero et al., 2018). Seasonal increases in the number of reported cases may be understood through two non–mutually exclusive frameworks. One is the Routine Activities Theory (Cohen & Felson, 1979), which suggests that warmer months may increase opportunities for IPV because couples spend more time in private settings with fewer third parties present, reducing informal social oversight from neighbors, coworkers or community members who might otherwise interrupt or deter violence. These seasonal shifts in daily routines increase the time perpetrators and survivors spend together in relative privacy, creating conditions consistent with the theory that help explain the observed summer peaks in police-reported IPV. A second framework is the General Affective Aggression Model (Anderson et al., 1995), which proposes that higher temperatures can elevate psychological arousal, stress, and irritability, increasing the likelihood that conflicts escalate into violence, which may also contribute to seasonal reporting patterns.

In addition to seasonal trends, police-reported IPV exhibited clear weekly rhythms, with the highest number of reports on Sundays, followed by Saturday and Friday, and the lowest reports mid-week (Tuesday through Thursday). These patterns are descriptive and align with previous observations in crime report data (A. M. Campbell et al., 2020; Grech & Burgess, 2011; Butke & Sheridan, 2010) and emergency department visits (Loder & Momper, 2020; Jónasdóttir et al., 2022), which also report weekend peaks in formal reporting of IPV. While the present data cannot identify causal factors, prior research suggests that weekend peaks in IPV reporting may be associated with factors such as increased household interaction, elevated interpersonal tension, higher alcohol consumption, accumulated stress over the workweek (Ivandić et al., 2024; Kusunoki et al., 2023; Sontate et al., 2021; VanBuren Trachtenberg et al., 2009), and increased participation in social gatherings. These findings are consistent with broader criminological and public health literature but remain hypotheses rather than conclusions derived from the current dataset.

### 4.1. Limitations and Strengths

This study provides valuable insight into IPV police reporting trends in Los Angeles, but several limitations should be acknowledged. The analysis relies on LAPD-reported crime data, which likely underrepresents less visible forms of IPV, including psychological, economic, and sexual abuse, as well as stalking. Moreover, police-recorded crime rates cannot capture the full scope of IPV because reporting varies by sex, age, ethnicity, income, and the survivor’s relationship to the offender (Decker et al., 2019).

In this study, cases were identified using the LAPD’s MO and Crime Type codes, providing a structured approach for capturing incidents that came to police attention. However, identifying IPV solely through these codes has inherent limitations: reporting practices may vary across officers and individual incidents, and subtle, chronic, or complex forms of IPV may not be fully documented. Cases were independently reviewed by a second coder with IPV expertise to reduce misclassification, yet residual errors or omissions due to the quality of the original reports cannot be ruled out, highlighting the structural limits of administrative data in fully representing survivor experiences.

It is also important to recognize that police-reported data inherently reflect reporting behavior rather than actual IPV incidence. Not all survivors contact law enforcement, and patterns in the data may be influenced by social, cultural, and institutional factors that shape help-seeking. Accordingly, observed trends, including seasonal or weekly variations, should be interpreted cautiously and cannot be assumed to indicate causal relationships or the true prevalence of IPV.

Despite these limitations, the study has several strengths. The use of a large, longitudinal dataset spanning four years enables the identification of seasonal, weekly, and long-term patterns in police reporting at the population level, even if individual incidents remain unreported. This temporal aggregation helps mitigate inconsistencies inherent in administrative data, providing insights into reporting dynamics rather than IPV occurrence. Time-series modeling serves as a supplementary tool to describe historical patterns in police-reported incidents and potential reporting trends, without implying a complete representation of IPV. Finally, reliance on publicly available LAPD Open Data ensures transparency and reproducibility, allowing other researchers to validate, replicate, or extend this work.

### 4.2. Implications for Policy and Practice

The findings of this study have broad relevance for multiple stakeholders involved in IPV prevention and response. Clinicians and healthcare providers can use temporal and demographic patterns to identify periods and populations with higher reporting, which may support targeted screening and trauma-informed care. Social service providers and community organizations can plan outreach, support programs, and emergency resources around peak reporting periods, such as weekends and summer months, to ensure timely availability of services. Policymakers can draw on these insights to allocate resources efficiently, design evidence-based prevention initiatives, and develop policies that reduce barriers to reporting and access to services. The observed seasonal and weekly patterns highlight the importance of considering temporal dynamics when interpreting police-reported IPV. While these patterns may reflect both reporting behavior and underlying IPV, they primarily inform administrative and service planning rather than casual conclusions about IPV occurrence. Recognizing these temporal patterns allows policymakers and practitioners to anticipate potential increases in reporting, even amid concurrent crises or emergency measures.

Although there are currently no documented examples of implementation specifically for IPV, evidence from other crime areas demonstrates how police data can inform multi-sector interventions. For example, the Cardiff Model in Wales (Shepherd et al., 1990) combines hospital and police data to identify interpersonal violence hotspots and guide targeted prevention efforts. Evaluations have reported reductions in hospital admissions for violence-related injuries and decreases in police-recorded assaults following its implementation (Florence et al., 2011). This example illustrates how systematic analysis of police-reported incidents, particularly when triangulated with data from emergency departments, community organizations, and other relevant sources, can support risk assessment, resource allocation, and coordinated action across sectors, suggesting potential pathways for translating insights from reporting data into practical strategies without implying causation.

## 5. Conclusions

Analysis of police-reported IPV incidents in Los Angeles indicates that reported cases predominantly involve younger adults and women, occur mainly in residential settings, and most incidents occur without a weapon, with non-physical forms underrepresented in the reporting data. Reports followed consistent seasonal and weekly cycles, with no evidence of a pandemic-specific shift. These observed reporting patterns provide actionable guidance for law enforcement and service providers to plan staffing, outreach, and prevention efforts during higher-reporting periods. Incorporating complementary data sources in future research could further illuminate underreported forms of IPV and support more effective interventions.

## Figures and Tables

**Table 1:** Descriptive Characteristics of IPV-related incidents reported to the Los Angeles Police Department between Jan 2020 and Dec 2023 (*N* = 74,776).

Variable	*N*	%
*Survivor Biological Sex*		
Female	57,838	77.35%
Male	16,938	22.65%
χ^2^	22,370.9 ^‡^	
*Survivor Age*		
18–24	12,789	17.10%
25–29	14,573	19.49%
30–34	13,969	18.68%
35–39	10,870	14.54%
40–44	7868	10.52%
45–49	5559	7.43%
50–54	3814	5.10%
55–59	2446	3.27%
60–64	1465	1.96%
65+	1423	1.90%
χ^2^	33,124 ^‡^	
*Incident Location*		
Residential	53,792	71.94%
Transportation	4873	6.52%
Retail	434	0.58%
Recreation/Entertainment	127	0.17%
Education	102	0.14%
Health and Care	138	0.18%
Religious	26	0.03%
Institutional/Public Service	146	0.20%
Public Space	13,625	18.22%
Other Business	987	1.32%
Other/Unspecified	526	0.70%
χ^2^	381,910.6 ^‡^	
*IPV Subtype*		
Physical IPV	57,589	77.02%
Psychological IPV	11,843	15.84%
Sexual IPV	3560	4.76%
Economic IPV	1342	1.79%
Stalking	442	0.59%
χ^2^	157,345.2 ^‡^	

^‡^ *p* < 0.001.

## Data Availability

The original data presented in the study are openly available from the LAPD Open Data portal at https://data.lacity.org/Public-Safety/Crime-Data-from-2020-to-Present/4372nrs-mtv8/about_data (accessed on 1 August 2025).

## Notes

1 Differences by descent (i.e., race and ethnicity) and reporting district were not examined due to the influence of systemic oppression, including racial bias, on police data collection and reporting.

2 The temporal restriction was applied due to temporary disruptions in data updates by the LAPD beginning in early 2024.

## Abbreviations

The following abbreviations are used in this manuscript:

IPV	Intimate Partner Violence
LAPD	Los Angeles Police Department
MO	Modus Operandi
SARIMA	Seasonal Autoregressive Integrated Moving Average
ARIMA	Autoregressive Integrated Moving Average
ACF	Autocorrelation Function
PACF	Partial Autocorrelation Function
STL	Seasonal-Trend Decomposition Using Loess
AIC	Akaike Information Criterion
